# Synergistic Effects of Tragacanth and Anti-ethylene Treatments on Postharvest Quality Maintenance of Mango (*Mangifera indica* L.)

**DOI:** 10.3390/plants12091887

**Published:** 2023-05-05

**Authors:** Emad Hamdy Khedr, Jameel Mohammed Al-Khayri

**Affiliations:** 1 Department of Pomology, Faculty of Agriculture, Cairo University, Giza 12613, Egypt; 2 Department of Agricultural Biotechnology, College of Agriculture and Food Sciences, King Faisal University, Al-Ahsa 31982, Saudi Arabia

**Keywords:** Keitt, fruit, coating, storage, edible film, anti-ethylene, melatonin

## Abstract

Mango (*Mangifera indica* L.) is one of the most popular tropical fruits grown in Egypt and several other countries, making it a potential export commodity. Excessive deterioration after harvest requires various treatments to maintain fruit quality. We evaluated the treatments effects of melatonin (MT) as an anti-ethylene agent and tragacanth gum (TRG) as an edible coating individually and together (MT–TRG) before storing mangoes at 12 °C for 32 days under 85–90% relative humidity. Compared with control, all treatments were significantly effective in preserving fruit quality. Fruits treated with MT–TRG showed significantly lower decay values, respiration rates, ethylene production, and weight loss than untreated fruits. MT–TRG treatment significantly enhanced fruit quality, thereby maintaining fruit appearance, flesh color, firmness, total soluble solids and phenolic contents, and pectin methyl esterase, polyphenol oxidase, and peroxidase activities during the storage period. We propose 200 µM MT + 1% TRG as a safe postharvest treatment to reduce the deterioration of mangoes and maintain fruit quality.

## 1. Introduction

Mango (*Mangifera indica* L.), a popular tropical and subtropical fruit, is considered a valuable fruit crop owing to its distinctive flavor and nutritional value [1]. Global mango consumption has increased in recent years [2]. Keitt is one of the most popular commercial mango cultivars planted in large areas. It has moderate peel thickness and rich aroma, with high total soluble solids (TSS) content and titratable acidity (TA) [3]. Unfortunately, a large amount of fruit is lost in the postharvest stage. Although mangoes are harvested at maturity, their storability is limited due to relatively high respiration levels. Additionally, fruits are exposed to numerous devastating effects, particularly when improperly handled [4]. Decay progresses for various reasons, including harvesting without relying on optimal maturity indices, poor handling, and using inappropriate temperature or humidity conditions during storage. These factors result in different physiological, physical, and pathological disorders of fruits [5].

Mangoes are climacteric fruits; ethylene can accelerate their ripening and senescence, leading to a short shelf life [3]. Various materials with different safety issues, such as 1-methylcyclopropene [6] and aminoethoxyvinylglycine [7], in addition to other compounds have been proposed as anti-ethylene agents [8,9]. Recently, melatonin (MT; N-acetyl-5-methoxytryptamine) has been widely recommended as an endogenous plant hormone essential for several different functions, such as plant organ development [10], fruit development, fruit ripening, and blooming [11]. It has also been shown to protect plants from various biotic and abiotic stresses [12]. Exogenous MT treatment has recently been reported as a successful postharvest treatment to reduce ethylene action, delay postharvest senescence, and improve chilling-injury tolerance in several fruits [13,14,15] by activating the antioxidant system, reducing postharvest deterioration, and maintaining fruit nutritional quality [16]. Moreover, Magri and Petriccione [17] revealed that treating fruits with MT during the pre-storage stage reduces deterioration and increases their antioxidant capacity. Further, Rastegar et al. [4] reported that MT content changes with fruit ripening. However, the relationships between MT content and the mechanisms regulating fruit ripening are complex. For example, during the ripening stage, MT content in berries decreases, whereas that in mango increases [4,17]. Such a variation requires further investigation to determine the effect of MT under each specific treatment.

Mangoes lose a large amount of water, leading to distinct weight losses. This constitutes an obstacle to transporting the fruit over long distances. Moisture loss is negatively reflected in the fruit in the form of wilting, significant weight loss, and decreased nutritional value [18]. Natural wax on the surface of the fruit keeps it shiny and maintains its permeability to water and gas. However, these waxes can be lost or damaged during fruit-handling procedures [19]. In this regard, fruit-coating films have received considerable attention in recent years. Edible, organic, biodegradable, and environmentally friendly coatings [20] are usually composed of polysaccharides, proteins, lipids, or a combination of these substances [21]. Wax is primarily used to reduce fruit weight loss and water loss from the fruit surface. Khedr [19] reported that coating treatments might improve fruit appearance, maintain fruit quality, and reduce fruit weight loss by approximately 50%. Li et al. [22] demonstrated that coating considerably reduces the respiratory rate, and waxed fruits exhibit a better physical appearance.

Tragacanth gum is a type of acidic polysaccharide that is derived from plants, with *Astragalus gummifer* being one of the most commonly used species for its secretion [23]. This biodegradable and biocompatible complex is classified as “generally recognized as safe” and is considered safe for use at levels ranging from 0.2% to 1.3%. Tragacanth gum has been applied to various commodities such as button mushroom, where it was used in combination with *Zataria multiflora* essential oil to delay browning and suppress microbial spoilage [24]. Additionally, treating harvested apricot fruits with tragacanth gum has been found to help modulate oxidative stress and maintain their quality [25].

However, there are limited studies regarding the effects of TRG and MT on postharvest quality characteristics of Keitt mangoes related to metabolic processes during cold storage and optimal storage conditions. Therefore, this study aimed to evaluate the effects of MT and TRG on Keitt mangoes in terms of delaying fruit ripening and senescence and maintaining quality characteristics under cold storage conditions.

## 2. Results

### 2.1. Rheological and Main Properties of TRG Edible Coatings

**Figure 1:**
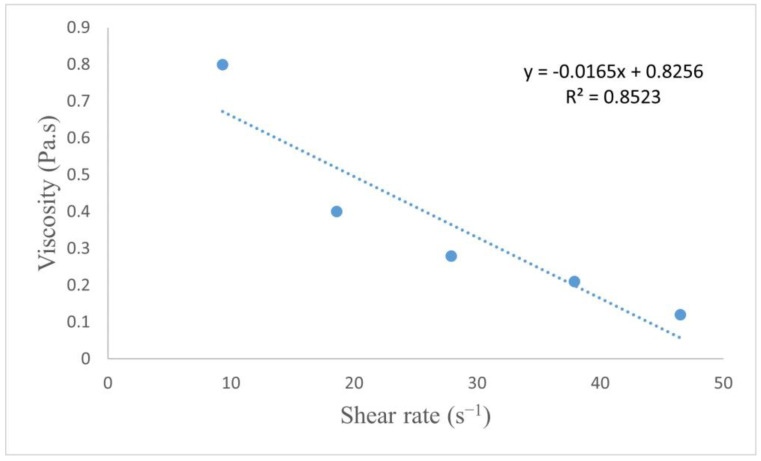
Tragacanth (TRG) film viscosity changes in response to different shear rates.

Figure 1 shows the relationship between viscosity and shear rate of TRG solutions. The solutions exhibited non-Newtonian fluid behavior, wherein the viscosity decreased with increasing shear rate. The TRG film had a viscosity of 0.4 Pa.s at room temperature. A previous study reported that the viscosity of 0.4 Pa.s was ideal for use in films [26]. Similar to the previous results by Mohammadifar et al. [27], the TRG film had good water solubility and color properties (Table 1). TRG treatment resulted in a glossy exterior of the fruit without any unwanted darkening or opaque appearance.

### 2.2. Coating Morphological Distribution on the Mango Surface

**Figure 2:**
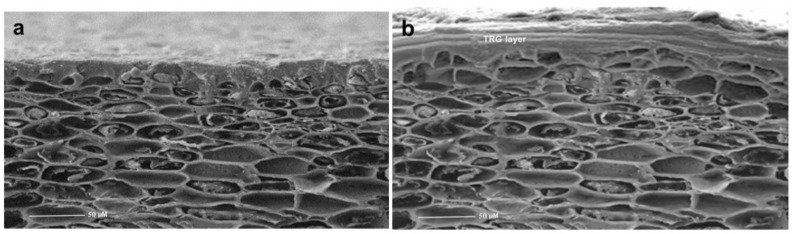
Images of fruit surface showing (**a**) uncoated healthy surface and (**b**) healthy surface coated with 1% TRG.

The TRG coating was clearly visible on the mango surface compared with the noncoated surface, as seen in Figure 2a,b. Furthermore, the flatness of the coatings altered in response to changes in the structure of the mango epidermis. This observation suggested that the coating was strongly bonded to the surface and that the coating formation and compactness were excellent. Furthermore, the surface had nearly no holes. This study also validated the coating’s high degree of adhesion to the mango surface, in agreement with Oak et al. [28].

### 2.3. Fruit Decay Percentage and General Appearance

**Figure 3:**
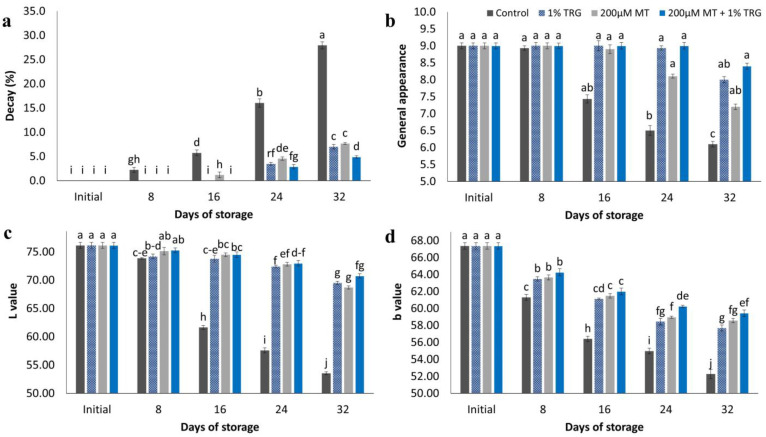
Effect of postharvest melatonin (MT) and tragacanth (TRG) treatments on the decay percentage (**a**), general appearance (**b**), *L* value (**c**), and *b* value (**d**) of Keitt mango fruits stored at 12 °C for 32 days. Vertical bars reflect the standard error of the means, with different letters indicating significant variance (*p* ≤ 0.05) across means, as determined by Duncan’s multiple range test.

Figure 3a shows the effects of MT and TRG treatments on the decay percentage of mango cv. “Keitt” during the 32-day cold storage period at 12 °C. The rate of deterioration increased continuously with prolonged storage. The differences between the treatments were significant. MT and TRG treatments were significantly effective in preserving fruit quality. Treatment with 200 µM MT + 1% TRG resulted in the lowest rate of deterioration (4.90%) after the 32-day storage period at 12 °C, whereas the rate of deterioration reached 27.94% in untreated fruits on the same day.

**Figure 4:**
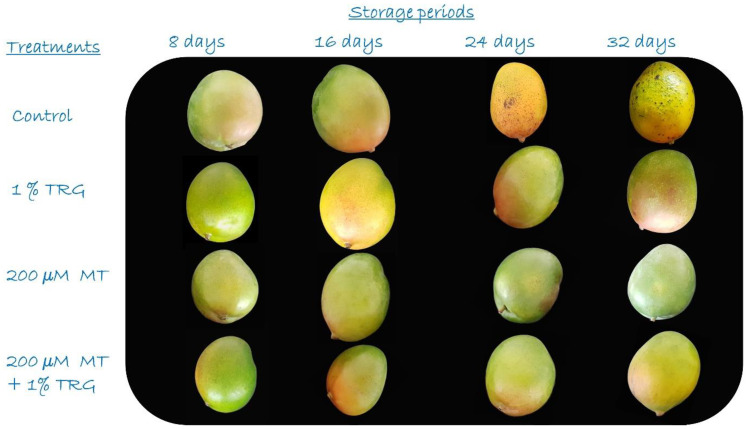
Effect of postharvest melatonin (MT) and tragacanth (TRG) treatments on the visual appearance of Keitt mango fruits stored at 12 °C for 32 days.

Figure 3b and Figure 4 present the effects of MT and TRG treatments on the general appearance of the fruit. In general, fruit quality decreased sharply after the 32-day storage period in untreated fruits, whereas treated fruits showed better quality scores. All TRG-treated mangoes were significantly more acceptable during the storage period and after the 32-day storage period at 12 °C. Treatment with 200 µM MT + 1% TRG and TRG alone maintained fruit appearance, as indicated by the scores of 8.40 and 8.33, respectively, whereas the control showed the lowest acceptability score at 6.10.

### 2.4. Flesh Color

Figure 3c,d show the effects of MT and TRG individually and together on Keitt mango flesh color based on the *L* and *b* values. One of the most prominent signs of mango ripening is a change in its color and softness after harvest [29]. The breakdown of chlorophyll and production of carotenoids are primarily responsible for this color change [30]. After the 32-day storage period at 12 °C, treatment with 200 µM MT + 1% TRG resulted in the highest pulp lightness (70.73), whereas the control pulp showed darkening (53.54). Further, fruits treated with 200 µM MT + 1% TRG showed the highest *b* value (59.43), whereas the control fruits showed the lowest *b* value (52.28) at the end of the storage period.

### 2.5. Weight Loss

**Figure 5:**
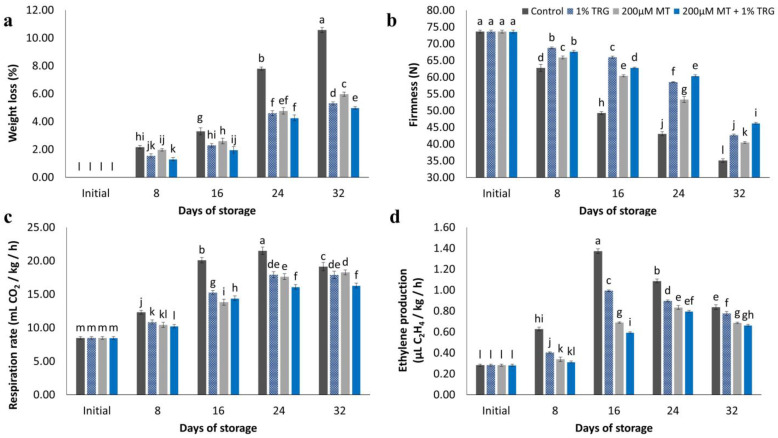
Effect of postharvest melatonin (MT) and tragacanth (TRG) treatments on the weight loss (**a**), flesh firmness (**b**), respiration rate (**c**), and ethylene production (**d**) of Keitt mango fruits kept at 12 °C for 32 days. Vertical bars reflect the standard error of the means, with different letters indicating significant variance (*p* ≤ 0.05) across means, as determined by Duncan’s multiple range test.

Weight loss increased consistently under all conditions during the 32-day storage period, as shown in Figure 5a. All treated fruits had lower weight loss values than untreated fruits. Control fruits showed approximately 50% higher weight loss than fruits treated with 200 µM MT + 1% TRG. The highest weight loss was detected in untreated fruits (10.57%) after the 32-day storage period at 12 °C, whereas fruits treated with 200 µM MT + 1% TRG and 1% TRG alone showed a weight loss of 4.98% and 5.30%, respectively.

### 2.6. Fruit Firmness

Flesh firmness is an important indicator of fruit quality. It is vital for fruit structure and handling processes as well as enzyme system processes for all fruit chemical contents. Fruit firmness gradually decreased with prolonged storage (Figure 5b). Fruit firmness was maintained under all treatments. Fruits treated with 200 µM MT + 1% TRG were significantly firmer than control fruits, which showed higher softening rates. At the end of the storage period, fruits treated with 200 µM MT + 1% TRG showed a firmness of 46.23 N, whereas control fruits showed a firmness of 35.07 N, as determined using previously reported by Silva et al. [31], Rastegar et al. [4], and Dong et al. [1].

### 2.7. Respiration Rate and Ethylene Production

Mangoes are climacteric fruits with a relatively high respiratory rate [32,33]. As shown in Figure 5c,d, MT and TRG treatments resulted in significantly lower respiratory rates than the control during cold storage at 12 °C. The respiratory rate increased significantly over time. Control and treated fruits showed a steady increase after 16 and 24 days, respectively. Subsequently, the respiratory rates continued to increase but not to the same degree. At the end of the storage period, fruits treated with 200 µM MT + 1% TRG showed the lowest respiratory rate (16.30 mL CO_2_/kg/h), whereas control fruits showed the highest respiratory rate (19.13 mL CO_2_/kg/h). The rate of ethylene production initially increased and then decreased. Peak ethylene production in MT-treated fruits was observed after 24 days, whereas that in control fruits was observed after 16 days. At the end of the storage period, fruits treated with 200 µM MT + 1% TRG showed the lowest ethylene production rate (0.66 µL C_2_H_4_ kg^−1^ h^−1^), whereas control fruits showed the highest rate (0.84 µL C_2_H_4_ kg^−1^ h^−1^).

### 2.8. TSS and TSS/TA Ratio

**Figure 6:**
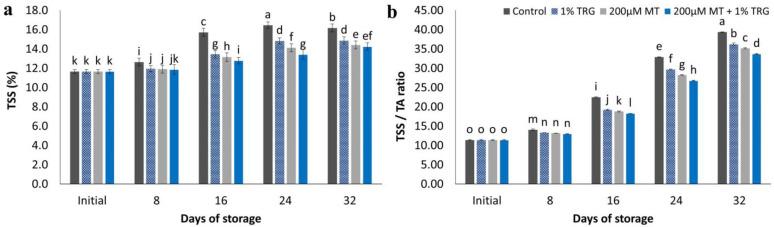
Effect of postharvest melatonin (MT) and tragacanth (TRG) treatments on the TSS (**a**) and TSS/TA (**b**) of Keitt fruit kept at 12 °C for 32 days. Vertical bars reflect the standard error of the means, with different letters indicating significant variance (*p* ≤ 0.05) across means, as determined by Duncan’s multiple range test.

Figure 6a shows the effect of different MT and TRG treatments on TSS content. Fruits treated with 200 µM MT + 1% TRG showed lower TSS contents than control fruits, which showed the highest contents during the cold storage period. A linear increase in TSS content was observed during the storage period at 12 °C. Control fruits showed the highest TSS content (16.17%), whereas fruits treated with 200 µM MT + 1% TRG showed the lowest TSS content (14.23%) at the end of the storage period. A similar trend was observed in TSS/TA ratio, which gradually increased during the storage period (Figure 6b). Control fruits showed the highest TSS/TA ratio (39.37), whereas fruits treated with 200 µM + 1% TRG showed the lowest ratio (33.71%) after the 32-day storage period under experimental conditions.

### 2.9. Total Phenolic Content

**Figure 7:**
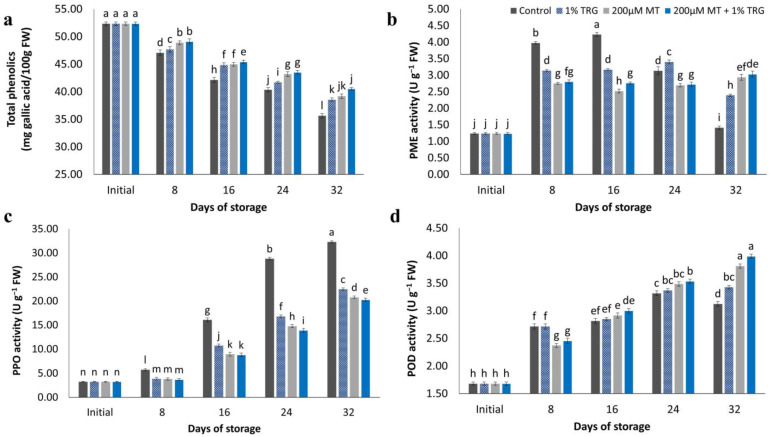
Effect of postharvest melatonin (MT) and tragacanth (TRG) treatments on the total phenolic content (**a**), pectin methylesterase (PME) activity (**b**), polyphenol oxidase (PPO) activity (**c**), and peroxidase (POD) activity (**d**) of Keitt mango fruits stored at 12 °C for 32 days. Vertical bars reflect the standard error of the means, with different letters indicating significant variance (*p* ≤ 0.05) across means, as determined by Duncan’s multiple range test.

Total phenolic contents decreased continuously during the storage period (Figure 7a). At the end of the storage period, control fruits showed significantly lower phenolic contents than fruits treated with 200 µM MT + 1% TRG. The ability of the fruit to maintain its nutritional value, color, and flavor as well as increased resistance and reduced disease occurrence relied on the total phenolic content [4]. The scavenging of reactive oxygen species by phenolic compounds is also crucial for increasing the antioxidant activity of fruits [34].

### 2.10. PME Activity Assay

The effect of different MT and TRG treatments on PME activity is shown in Figure 7b. Fruits treated with 200 µM MT + 1% TRG showed the lowest mean activity, whereas untreated fruits showed the highest values during the cold storage period. A fluctuation trend in PME was observed during the storage period at 12 °C. At the end of the storage period, control fruits showed the lowest PME activity (1.41 U g^−1^ FW), whereas fruits treated with 200 µM MT + 1% TRG showed the highest activity (3.03 U g^−1^ FW).

### 2.11. Effects of MT and TRG on PPO and POD Activities

The effect of MT and TRG treatments on PPO activity is shown in Figure 7c. Fruits treated with 200 µM MT + 1% TRG showed the lowest mean activity, whereas control fruits showed the highest values during the cold storage period. A linear increase in PPO activity was observed during the storage period at 12 °C. At the end of the storage period, control fruits showed the highest PPO activity (27.53 U g^−1^ FW), whereas fruits treated with 200 µM MT + 1% TRG showed the lowest activity (20.25 U g^−1^ FW). The effect of different MT and TRG treatments on POD activity is shown in Figure 7d. Fruits treated with 200 µM MT + 1% TRG showed the lowest mean activity, whereas control fruits showed the highest values during the cold storage period. A linear increase in POD activity was observed during the storage period at 12 °C. At the end of the storage period, control fruits showed the lowest POD activity (3.12 U g^−1^ FW), whereas fruits treated with 200 µM MT + 1% TRG showed the highest activity (3.99 U g^−1^ FW).

### 2.12. Correlation between Different Quality Attributes

**Figure 8:**
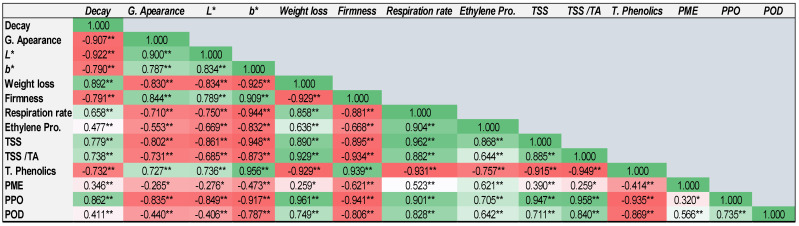
Correlation matrix among several quality attributes of Keitt mango fruits stored at 12 °C for 32 days in response to melatonin (MT) and tragacanth gum (TRG) treatments. Asterisks (* or **) denote statistically significant difference at *p* ≤ 0.05 or 0.01, respectively, in the Pearson correlation analysis, which was performed with three replicates (n = 3).

The correlation matrix among various quality attributes of Keitt mango fruits under experimental conditions is shown in Figure 8. Most of the observed values were statistically correlated with each other. Significant correlations were observed among weight loss, respiratory rate, ethylene production, deterioration rate, and PPO, POD, and PME activities. Fruit firmness was significantly negatively correlated with respiration rate, deterioration rate, ethylene production, weight loss, and PPO, POD, and PME activities. Further, it was positively correlated with color, appearance, and total phenolic content. These results are in agreement with those of previous studies [3,4,5,33,35,36].

## 3. Discussion

Control of fruit respiration and water loss from fruit is central to several physiological and biological actions, which will finally translate into different characteristics for fruit quality and also determine the longevity of these fruits. TRG film has a viscosity of 0.4 Pa.s when measured at room temperature, and this viscosity was obtained in early investigations to be ideal for use in fruit films [26]. Similar to the previous study by Mohammadifar et al. [27], TRG film had good water solubility as well as good color properties. TRG treatment resulted in the fruit having a glossy exterior without any unwanted darkening or opaque appearance. The coating was clearly visible on the mango surface compared with the noncoated surface, as seen in Figure 2a,b. Furthermore, the flatness of the coatings altered in response to changes in the structure of the mango epidermis. This observation suggested that the applied coating was strongly bonded to the surface and that the coating formation and compactness were excellent. Furthermore, the surface had nearly no holes. This study also validated the coating’s high degree of adhesion to the mango surface, in agreement with Oak et al. [28].

Fruit development is characterized by significant changes. When climacteric fruits such as mangos ripen, the respiration rate rapidly increases [37] and undergoes significant metabolic changes [34]. In general, ethylene is believed to enhance signal transmission. In response to several signals, it promotes the expression of genes that encode enzymes responsible for changes in color, flavor, and texture during ripening [32]. Some anti-ethylene compounds such as salicylic acid and 1-methylcyclopropene inhibit ethylene synthesis and reduce ethylene production. Further, endogenous ethylene inhibitors limit hormonal activity by competing with ethylene for ethylene receptors [38]. Additionally, ethylene can be oxidized to CO_2_ using exogenous scavenging agents such as potassium permanganate and ozone [39]. In addition, coating is considered as a modified atmospheric tool. In addition to its role as a semipermeable barrier to moisture, solvents, and gases, coating slows down the rates of oxidation, respiration, and weight loss [40]. At the end of the storage period, control fruits had a higher decay rate than treated fruits. The edible coating acts as a physical barrier to oxygen, thereby slowing down the respiration of the fruit [36]. Thus, the hydrolytic enzymatic activity was reduced, and softening of the fruit hampered the decrease in the respiration rate. TRG-based coating acts as a semipermeable barrier, restricting gas exchange and forming a superficial barrier layer on the fruit. Therefore, the nonenzymatic antioxidant capacity of the fruit was preserved throughout the storage period (ethylene, CO_2_, and oxygen), consistent with the results of previous studies [13,14,37]. Reduced oxygen permeability in TRG-coated fruit inhibits the activity of enzymes in bioactive chemical oxidative reactions, causing significant changes in respiration pathway metabolism [41] and slowing down the ripening process due to the inhibition of ethylene production [42]. Both individual and combined treatments of MT and TRG have the potential to maintain the external appearance of the fruit, which has remained acceptable for marketing to consumers for a prolonged period. The findings are closely related to the characteristics of the film and the extent to which the destruction progresses inside the fruit, consistent with the results reported by Dong et al. [1]. According to the results, deterioration significantly increases during cold storage, and mangoes are climacteric fruits with increased metabolic activity, ethylene production, and respiration, all of which accelerate ripening after harvest. These processes are related to fruit weight loss, rapid softening owing to decomposition, and peel browning, all of which diminish storability [1]. The capacity of the TRG coating to delay the ripening of mangoes may improve the resistance of fruits to pathogen invasion during storage. Furthermore, the antimicrobial properties of TRG are also important [43]. The results were similar to those of a previous study by De Oliveira et al. [44], in which MT slowed down biological processes and prevented several diseases. Additionally, Bhardwaj et al. [45] reported that MT helps reduce decay, thereby markedly decreasing the prevalence and severity of numerous physiological problems and storage disorders. Accordingly, the cumulative effect of 200 µM MT + 1% TRG significantly reduced the fruit loss rates under the experimental conditions.

Flesh color is one of the primary characteristics that represents the distinguished fruit quality. Therefore, when determining mango fruit maturity or quality indices, it is crucial to estimate the color. In general, mango flesh color is a more reliable index than peel color [34]. All fruits showed a continuous increase in yellow color intensity with ripening. In this regard, the *L* and *b* values decreased with a darker yellow color. All treatments showed late flesh-color changes compared with the control, in accordance with the findings reported by Kittur et al. [46]. Liu et al. [41] also reported a parallel relationship between fruit ripening and mesocarp-color changes. Mango fruit ripening involves a series of biochemical responses that follow the development of pigments via carotenoid biosynthesis. The modified internal environment of the coated fruits may be responsible for late ripening, which reduces chlorophyll degradation and carotenoid biosynthesis [47]. Polysaccharide-based composite coats have a synergistic effect on color retention by enhancing the development of coloring pigments in mango fruit [48]. The findings showed that MT and TRG treatments inhibited flesh and pulp discoloration.

Determining fruit weight loss is an indicator of fruit quality and freshness. Fruit weight loss primarily occurs due to water loss during respiration and transpiration [18]. The negative impact of weight loss is not only related to economic loss but also to reduced water content, resulting in adverse changes such as fruit exocarp shrinkage and decreased nutritional value. The characteristic texture of the peel and pulp is lost due to cellular breakdown [3]. In addition, various metabolic processes that occur after harvest and during storage are significantly associated with water loss. Several studies have revealed that the respiration rate is a critical factor related to weight loss [19]. The direct effects of MT and TRG on respiration rates can lead to reduced water loss. Additionally, as TRG closes several small pores, it is a vital barrier to water loss (evaporation) from the fruit. It also directly affects cell turgidity in fruit peels [49]. In this regard, the effects of storage conditions (temperature and humidity) must be considered. Liu et al. [41] and Kassem et al. [36] reported similar results in Guifei and Tommy Atkins cultivars.

Physical changes in fresh mango fruits, such as softening, are essential for fruit handling, storage, and consumer acceptance [48]. The coating film acts as an additional external wall for the fruit, thereby maintaining the cell wall structure and closing the small pores in the exocarp. This role is also exhibited by MT for reducing respiration rates and minimizing cell wall hydrolytic enzymatic activities [5,49], particularly pectin methylesterase activity, which correlates with fruit softening [50]. According to Zhang et al. [51], the effects of mango pectin methylesterase and polygalacturonase on firmness are primarily attributable to changes in the cell wall structure and disintegration of various cell wall components. Throughout fruit ripening, PME demethylates pectin, allowing polygalacturonase to hydrolyze the galacturonic acid chain of pectic acid, resulting in the disintegration of the colloid layer in the cell wall and fruit softening [52]. TSS represents one of the highest quality attributes in relation to the consumer. Actually, TSS values are significantly influenced by carbohydrate changes in response to several related enzyme activities. The primary indicator for flesh flavor that expresses the results of many biological processes is the ratio of TSS to acid [31]. Occasionally, focusing just on the value of one of them may give a false idea about the quality and flavor of fruit. However, persistent ripening during cold storage is most likely the cause of the increase in TSS and TSS/TA during storage and the decrease in TA. The decrease in acidity due to consumption of organic acids in the fruit during respiration and biological changes in starch, glucose, and sucrose during ripening can be attributed mainly to higher total soluble solids to acid ratio, but total soluble solids decrease with prolonged storage [3]. The late increase of TSS in the treated fruit, especially in the MT treatments compared to the control group, is mostly related to the effect of these treatments on ethylene production and respiration rate [31,53]. Although both MT and TRG exert effects on ethylene production and respiratory rate, the results revealed that MT has a more prominent role in this regard, affecting TSS content. Rastegar et al. [4] reported similar results during mango storage at 15 °C for 4 weeks. The ability of the fruit to maintain its nutritional value, color, flavor, increased resistance, and reduced disease occurrence depends on its total phenolic content [4]. The scavenging of reactive oxygen species by phenolic compounds is also crucial for increasing the antioxidant activity of fruit [34]. The concentration of these phenolics decreased due to the disintegration of fruit cell components caused by PPO activity after ripening [4]. The highest phenolic content (40.51% mg gallic acid/100 g FW) was observed in fruits treated with 200 µM MT + 1% TRG. In contrast, control fruits showed the lowest phenolic content (35.62% mg gallic acid/100 g FW) at the end of the storage period. Rastegar and Atrash [34] reported a similar trend. In this regard, MT and TRG, which activate antioxidant enzymes and maintain the firmness of the cell tissue, could limit the increase in PPO activity in treated fruits. The current findings are consistent with those of the study by Rastegar et al. [4], which revealed that high concentrations of MT maintain the total phenolic content in mango fruit under cold storage conditions. Moreover, Kassem et al. [36] reported similar findings in Tommy Atkins mangoes.

PME activity decreased continuously during ripening in both control and MT-treated fruits, although the reduction was significantly less in MT-treated fruits, in line with a previous study [41] on Guifei mango fruit using postharvest MT application. PME activity significantly decreased after harvest. However, softening is a complicated process primarily initiated by alterations in the cell wall caused by breakdown of various cell wall components [1]. PME acts with other enzymes such as polygalacturonase and β-galactosidase to depolymerize and dissolve the pectin matrix in the cell wall [47]. Treated mango fruits had a slower decline in PME activity. The maintenance of fruit firmness can be explained by the fact that removing more methyl groups from polygalacturonic acid under high PME activity conditions can increase the number of binding sites that could interact with free calcium, thus increasing cell wall rigidity [51]. PME demethylates pectin during the ripening process of the fruit, facilitating the hydrolysis of the galacturonic acid chain of pectic acid [54], which dissolves the colloidal layer in the outer layer and leads to the softening of the fruit [52]. Because of decreased PME activity during storage, MT and TRG coatings reduced the concentration of polygalacturonase substrates, reduced pectin decomposition, and maintained mango firmness, consistent with the findings reported by Sajid et al. [55]. Fruit polyphenol content is mainly converted by PPO to quinones, followed by polymerization to form melanin, which eventually results in discoloration and reduced fruit quality [56]. PPO activity in all fruits increased gradually throughout the storage period, except in the control, in which it decreased at the end of the storage period. However, MT and TRG treatments constantly maintained PPO activity at a reasonably low level (Figure 7c), indicating that MT and TRG can diminish PPO activity, in accordance with the findings of Dong et al. [1] and Yu et al. [43]. POD is an essential enzyme in fruits, and reducing oxidation can help restore ascorbate and glutathione metabolites. Under experimental conditions, the activity of POD gradually increased (Figure 7d) and then decreased. Likewise, Chen et al. [57] and Yu et al. [43] reported that coating increased POD activity and decreased PPO activity, thereby decreasing postharvest loss and improving the storability of Xinyu tangerines and mango. Bhardwaj et al. [14] found similar results with MT treatment. Moreover, Wei et al. [58] and Javed et al. [59] reported similar POD activity. These results indicate that the use of 200 µM MT + 1% TRG showed promising complementary effects to preserve fruit quality, and it is considered an excellent, promising, and safe alternative treatment to many postharvest fruit treatments.

## 4. Materials and Methods

### 4.1. Fruit Material and Chemicals

Mango fruit cv. “Keitt” (*M. indica* L.) were harvested at a fully mature stage from 10-year-old trees, which were planted 2 × 3 m apart and grown in sandy soil with a drip irrigation system in the Beheira Governorate of Egypt (30°16′31.8′′ N, 30°32′16.4′′ E). Fruits were harvested depending on the color and firmness of the flesh, based on the indices reported by Ngamchuachit et al. [60]. The mangoes were immediately transported to the postharvest laboratory at Agricultural Systems Development Unit, Faculty of Agriculture, Giza, Egypt. In total, 360 mangoes of uniform size, color, and quality were selected and randomly divided into four groups of 90 mangoes. All selected mangoes were immersed in a disinfectant solution containing 0.05% sodium hypochlorite (pH 7) for 3 min. Next, the fruits were rinsed with water and air dried. Only reagent-grade chemicals were used in this study. The applied concentrations and immersion durations of MT and TRG (Sigma-Aldrich Inc., St. Louis, MO, USA) were based on previously reported pretreatment screening and studies.

### 4.2. Postharvest Treatments and Application Procedures

To prepare the coating solution, TRG gum powder obtained from Sigma-Aldrich (St Louis, USA) was added to 1 L of sterile double-distilled water in a beaker. The mixture was then homogenized thoroughly on a magnetic stirrer at 70 °C for 3 h. The resulting homogenate was stored at 5 °C for 24 h to allow for complete hydration of the TRG. Subsequently, a surfactant and plasticizer were added to the coating solution in the form of Tween-20 (0.25%) and glycerol (1%), respectively. The coating solutions were blended in a blender for 5 min and kept at room temperature for 60 min to eliminate any air bubbles [25].

The first group of fruit was immersed in distilled water for 120 min (control). The second group was immersed in 200 μM MT for 120 min at 25 °C under low-light conditions. The third group was immersed in 200 μM MT for 120 min, followed by treatment with 1% TRG for 5 min. The fourth group was only treated with 1% TRG. All mangoes were left to dry at room temperature for 2 h, packaged in cartons, and stored at 12 °C under 85–90% relative humidity for 32 days. Fruit quality was determined under each treatment repeatedly, with fruit storage intervals of 8 days during the 32-day storage period. Three fruits were used in each of the three replicates to evaluate all parameters.

### 4.3. Characterization of the TRG Film

#### 4.3.1. Rheological and Main Properties of TRG Edible Film

Differences in temperatures and the rheological properties of TRG (shear rate and viscosity) were evaluated using a rheometer (Brookfield Engineering laboratories DV-III Ultra, AMETEK Brookfield, Middleboro, MA, USA) according to C’aceres et al. [26]. The samples were arranged in a tiny sample adaptor, and the desired temperature was maintained using a constant temperature water bath. The SC4-21 spindle was chosen for the test, and the viscometer’s operating range was between 10 and 50 rpm. Using a Minolta CR-400 chroma meter (Minolta, Osaka, Japan), the color parameters of the film were determined: lightness (L*, light to dark direction), redness (a*, red to green direction), as well as yellowness (b*, yellow to blue direction). Index of total color difference (ΔE*) was calculated as following: ${\Delta E}^{*}=\sqrt{{\left({\Delta L}^{*}\right)}^{2}+{\left({\Delta a}^{*}\right)}^{2}+{\left({\Delta b}^{*}\right)}^{2}}$, where ΔL*, Δa*, and Δb* are the variances between the consistent color value of the film and the white standard (L*, a*, and b* equal 93.60, 0.95, and 0.46, respectively), according to Pathare et al. [61]. TRG film thickness (mm) was determined using a digital caliper.

Mohammadifar et al. [27] described a process for determining humidity content and solubility; film samples (1 × 5 cm) were weighed and then dried in an oven at 105 ± 1 °C until they reached a constant weight (W_1_). The moisture content was estimated using the reduced weight. The dried films were immersed in 25 mL deionized water for 24 h at 25 °C with constant stirring to test their water solubility (50 rpm). To calculate the weight (W_2_) of the insoluble contents, the undissolved materials were separated by filtering and dried at 105 °C in the same manner. Finally, the water solubility was calculated using the following formula: $Water\;solubility\left(\%\right)=\frac{W1-W2}{W1}\times 100$.

#### 4.3.2. Surface Morphology of Coating on the Mango Surface

Differences in microstructure between coated and non-coated mango were analyzed through SEM analyses. The mango peels of the test fruits were cut randomly using a stainless-steel blade and completely dried by vacuum freezing. Samples were examined using a JSM-6000F scanning electron microscope (JEOL, Tokyo, Japan), according to Quirós-Sauceda et al. [62].

### 4.4. Postharvest Treatment Effects on Mango Quality Characteristics

#### 4.4.1. Decay Percentage

Any symptoms of fruit deterioration (physiological or pathological disorders) during storage were evaluated and excluded. The decay percentage was calculated as the ratio of the number of deteriorated fruits to the total number of fruits at the beginning of storage × 100.

#### 4.4.2. Fruit General Appearance

Appearance quality was visually assessed using the visual score reported by Mitcham et al. [63]. On a scale of 1–9, 1, 3, 5, 7, and 9 referred to unacceptable, poor, fair, good, and excellent, respectively.

#### 4.4.3. Flesh Color

A Minolta CR-400 chroma meter (Minolta, Osaka, Japan) was used to measure the L* and b* values objectively. These values were based on the CIE L* a* b* concept on both side cheeks of each fruit [61].

#### 4.4.4. Weight Loss

The mangoes were weighed using a digital balance at the beginning of storage and on each investigation day. This procedure was used to calculate the weight loss percentage by subtracting the final weight from the starting weight [64].

#### 4.4.5. Fruit Firmness

According to Mitcham et al. [63], flesh firmness was assessed on both pared surfaces of each fruit using an 8 mm diameter fruit pressure tester probe (Mecmesin, force-torque test, England). The data are presented as N.

#### 4.4.6. Respiratory Rate and Ethylene Production

The fruit respiratory rate and ethylene production were estimated by incubating mangoes in airtight 4 L glass containers for 24 h under the same experimental conditions and using gas chromatography for CO_2_ analysis (Servomex 1400, Washington, DC, USA). The respiratory rate was reported as ml CO_2_/kg/h. Gas samples for ethylene production were obtained from the same containers following the same method using an ethylene analyzer (Model F-950; Three Gas Analyzer, Felix Instruments, Camas, WA, USA). Ethylene production was expressed as µL C_2_H_4_ kg^−1^ h^−1^ [65]. The respiratory rate and ethylene production were calculated as follows:

$$Respiratory\;rate=\frac{carbon\;dioxide\;produced\times head\;space\;volume}{fruit\;weight\times incubation\;period}$$

$$Ethylene\;production=\frac{ethylene\;produced\times head\;space\;volume}{fruit\;weight\times incubation\;period}$$

#### 4.4.7. TSS and TSS/TA

TA was assessed in accordance with A.O.A.C. guidelines [66]. The results were expressed as a percentage of citric acid, the most dominant organic acid in mango fruit (g citric extract/100 g fresh weight). TSS content was assessed using drops of juice and PAL-1 digital refractometer (ATAGO, Co., Ltd., Tokyo, Japan).

#### 4.4.8. Total Phenolic Content

Total phenolic content was determined based on the Folin–Denis reaction method [67] at a wavelength of 765 nm. It was calculated based on the standard curve of known gallic acid concentrations, and the results are expressed as mg gallic acid/100 g FW.

#### 4.4.9. Pectin Methylesterase (PME) Activity Assay

According to a previously published procedure used to measure PME activity (E.C. 3.1.1.11), 4 g of mango sample was ground using 9% (*w*/*v*) NaCl solution before centrifugation for 30 min after shaking for 4 h [68]. The PME extract was obtained from the supernatant and then mixed with 0.75 mL of water, 2.0 mL of pectin solution, and 0.15 mL of bromothymol blue. The results were obtained at a wavelength of 620 nm and expressed as U/g FW.

#### 4.4.10. Polyphenol Oxidase (PPO) and Peroxidase (POD) Activity Assays

The enzyme extraction and assay were conducted based on the methods reported by Zhou et al. [69] and Yu et al. [43]. A mixture of 0.1 mol L^−1^ phosphate buffer (1 mol L^−1^ NaCl and 2% (*w*/*v*) polyvinylpolypyrrolidone; pH 6.4) and 1% Triton X-100 (*v*/*v*) was used to isolate enzymes. Briefly, 10 g of mango fruit sample was mixed with 10 mL of enzyme extraction solution. After 3 min of homogenization, the mixture was centrifuged for 20 min at 14,000 rpm and 4 °C. The enzyme activity was assessed by analyzing the clear supernatant. To assess PPO activity (E.C. 1.14.18.1), 0.2 mL of the enzyme extract was mixed with 3.2 mL of phosphate buffer (0.1 mol L^−1^, pH 5.5) containing 0.06 mol L^−1^ catechol. The absorbance was measured at 420 nm for 1 min, and 1 unit of PPO activity was defined as the amount of enzyme causing a 0.01 unit rise in absorbance per min. The results were represented as U/g FW. For POD activity (E.C. 1.11.1.7), the reaction mixture contained 0.1 mL of the enzyme extract, 0.5 mL of 0.03 mol L^−1^ guaiacol, 0.2 mL of 0.5 mol L^−1^ H_2_O_2_, and 2.7 mL of phosphate buffer (0.1 mol L^−1^, pH 5.5). The absorbance was measured at 470 nm for 5 min, and 1 unit of POD activity was defined as the amount of enzyme causing a 0.01 unit rise in absorbance per min. The findings are expressed as U/g FW.

### 4.5. Statistical Analysis

This study was planned as a completely randomized design with three replicates. Data were analyzed via two-way analysis of variance using MSTAT-C software package to determine the effects of treatments, storage time, and their interaction. All tests were performed in triplicate. Duncan’s multiple range test was used to assess significance. *p*-values of ≤0.05 were considered to indicate statistical significance [70]. Pearson’s correlation analysis was also performed to examine the relationships between the mango quality parameters during storage at *p*-values of ≤0.05 and ≤0.01.

## 5. Conclusions

This study focused on the relationship between MT and TRG treatments and mango fruit quality during storage at 12 °C under 85–90% relative humidity for 32 days. Several quality parameters measured in Keitt mango fruits that were preserved under MT and TRG treatments by reducing the rate of ripening-related changes that led to deterioration. These treatments reduced the distinct rise in fruit respiratory rates and weight loss during the 32-day storage period. Compared with control, all treatments were significantly effective in maintaining the fruit quality and had a significant positive influence on TSS and phenolic contents, decay percentage, fruit softening, undesirable color changes, and PPO and POD activities during the storage period. The use of 200 µM MT + 1% TRG showed promising complementary effects to preserve fruit quality and is considered an excellent, promising, and safe alternative to several postharvest fruit treatments.

## Figures and Tables

**Table 1:** Primary properties of the applied tragacanth edible film.

Thickness (mm)	Water Content (%)	Water Solubility (%)	L*	a*	b*	ΔE
0.08	23.91	14.67	84.74	0.61	10.02	9.97

## Data Availability

The original contributions presented in the study are included in the article, and further inquiries can be directed to the corresponding authors.
